# Approaching high-performance of ordered structure Sb_2_Te_3_ film via unique angular intraplanar grain boundaries

**DOI:** 10.1038/s41598-020-63062-z

**Published:** 2020-04-06

**Authors:** Ming Tan, Liyu Hao, Hui Li, Cong Li, Xiaobiao Liu, Dali Yan, Tie Yang, Yuan Deng

**Affiliations:** 1 College of Science, Henan Agricultural University, Zhengzhou, 450002 China; 2 School of Physical Science and Technology, Southwest University, Chongqing, 400715 China; 3 College of Physics and Materials Science, Tianjin Normal University, Tianjin, 300387 China; 4 Beijing Key Laboratory of Special Functional Materials and Films, School of Materials Science and Engineering, Beihang University, Beijing, 100191 China

**Keywords:** Structural properties, Nanoscale materials

## Abstract

In this paper, we present an innovative electric-field-assisted magnetron-sputtering deposition method for films preparation. By grain boundary-engineering, we successeful obtained the ordered Sb_2_Te_3_ film with greatly high figure of merit via controlling external electric field. It has been found that the electric field can induce the change in the angle of intraplanar grain boundaries between (0 1 5) and (0 1 5) planes, which leads to the enhanced holes mobility and maintained low thermal conductivity. The energy filtering takes place at the angular intraplanar grain boundaries. At room temperature, a high *ZT* value of 1.75 can be achieved in the deposited Sb_2_Te_3_ film under 30 V external electric field. This is a very promising approach that the electric field induced deposition can develop high-performance Sb_2_Te_3_-based thermoelectric films.

## Introduction

The thermoelectric (TE) materials have drawn increasing attentions due to direct conversion between electrical and thermal energy. TE property of materials is determined by the dimensionless TE figure-of-merit (*ZT* = *S*^2^*σT/*κ), which can be enhanced by optimization of thermal conductivity (*κ*), Seebeck coefficient (*S*), and electrical conductivity (*σ*) at a Kelvin temperature (*T*)^1–5^. However, simultaneous optimization is challenging because it is very difficult to control the three interrelated TE properties independently. To increase *ZT* value, many scientists have been devoted into overcoming conventional *κ*-*σ* and *σ*-*S* trade-off in recent decades^6–9^. Theoretical and experimental results have shown that transport properties of phonon and electron can be greatly optimized by the low-dimensional structure^10–12^.

For decades, Bi_2_Te_3_ and Sb_2_Te_3_-based materials have been excellent candidates for cooling applications and power generation due to their high *ZT* values at low-temperature. Recent studies have shown that property enhancement can be obtained by controlling structure growth^13^. Our previous works demonstrate that a preferential way can be constituted via ordered-structuring, promoting carrier transport and carrier mobility in Bi_2_Te_3_-based films, thus enhancing *ZT* values^14,15^. In this work, we further realize accurate grain boundary-engineering to achieve ordered structure Sb_2_Te_3_ films using external electric field. Sb_2_Te_3_-based nanostructures have been synthesized by various methods including thermal evaporation, solvothermal, and electrochemical deposition, etc^16–22^. These methods have many advantages. But these methods all are difficult to accurately control over grain boundary-engineering in large scale. To overcome this challenge, the deposition substrate was applied external electric field. Therefore, the deposition of Sb_2_Te_3_ films can be tuned by adjusting the external electric field, leading to excellent carrier mobility (*μ*) and *κ*. Subsequently, a high *ZT* value of 1.75 for the Sb_2_Te_3_ film can be achieved at room-temperature.

Hence, this work aims to control grain boundary-engineering for Sb_2_Te_3_ films. Simultaneously, our goal here is to understand the relation between grain boundary-engineering and electric field. For the first time, a simple in-plane electric-field-assisted deposition technique is carried out for Sb_2_Te_3_ films. We demonstrate that the interrelationship between the grain boundary and the property of films can help to better understand grain boundary-engineering of this kind of material. This work reveals the importance of grain boundary-engineering that is realized by simply optimizing electric field. Furthermore, it provides a new way to control the structural configuration of thin-film materials with possible relevance to enhancement of performance.

## Results and discussion

**Figure 1:**
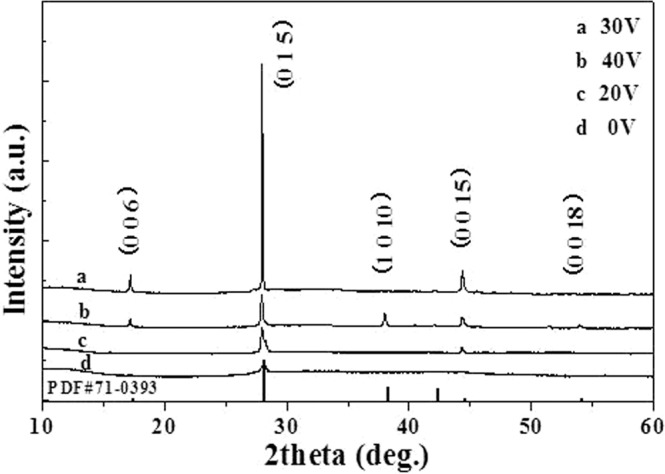
XRD patterns of Sb_2_Te_3_ films deposited under different electric fields (0, 20, 30 and 40 V).

In order to collect details of the phase structure, films are examined by XRD. Figure 1 presents XRD patterns of Sb_2_Te_3_ films prepared by assisted voltages of 0 V, 20 V, 30 V, and 40 V. As shown in Fig. 1, the disordered structure Sb_2_Te_3_ film prepared under no assisted voltage shows an approximate amorphous structure. Only a very weak (0 1 5) peak of the film appears. When the assisted voltage becomes large to 20 V, an obvious (0 1 5) peak and a weak (0 0 15) texture appear in the film. With increasing the assisted voltage to 30 V, (0 0 6) peak appears and (0 0 15) texture becomes strong, and a greatly preferential orientation (0 1 5) peak is observed in the ordered structure Sb_2_Te_3_ film. A single Sb_2_Te_3_ phase, consistent with the standard card (PDF#71-0393) of Sb_2_Te_3_, is obtained from the sample. However, the intensity of (0 0 6), (0 1 5), and (0 0 15) textures become weak compared with the ordered structure film, while obvious (1 0 10) and (0 0 18) peaks are found in the film fabricated by the voltage of 40 V. The grain size can be estimated from XRD patterns by Scherrer equation^23^, *D* = 0.89*λ*/(*β*cos*θ*), where *λ* = 1.54056 Å, *θ* is the angle of Bragg reflection, *β* is full-width at half-maximum (FWHM) corresponding to (0 1 5) peak. Table S1 shows the grain sizes of Sb_2_Te_3_ films in the Supporting Information. It is noted that the grain size is enhanced with increasing external electric field. It confirms that the in-plane electric field induces the crystal structure change. The growing grains can be sufficiently mobile, under the effect of electric field, to migrate to the sites for crystallization growth. Also, this implies that the proper intensity of electric field is responsible for the growth of the ordered structure Sb_2_Te_3_ film.

**Figure 2:**
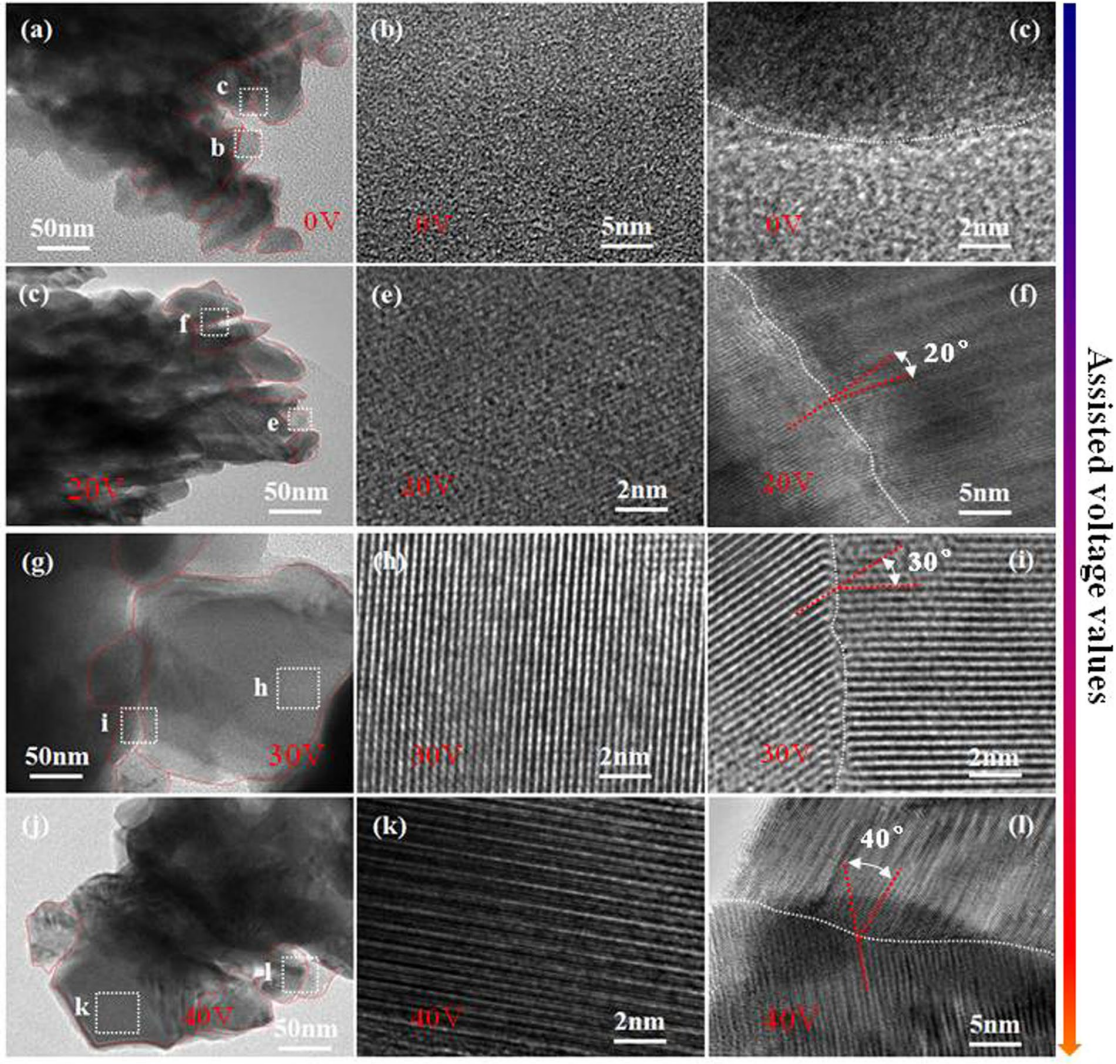
**(a,d,g,j**) TEM images of Sb_2_Te_3_ films prepared by assisted voltages of 0 V, 20 V, 30 V, and 40 V, respectively, (b,c; e,f; h,I; k,l) HRTEM images of the selected area correspondingly marked by the squares in (**a,d,g,j**). (A interface between both nanoparticles is located at the dotted curve.).

**Figure 3:**
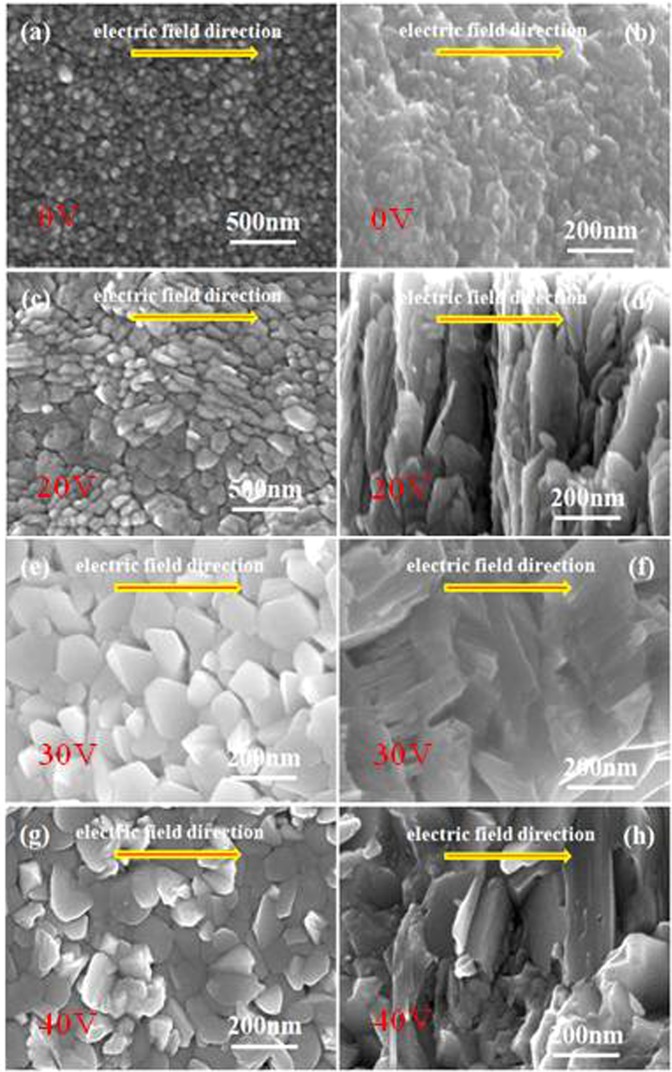
Surface view and cross-sectional view of Sb_2_Te_3_ films prepared by assisted voltages of (**a,b**) 0 V, (**c,d**) 20 V, (**e,f**) 30 V and (g,h) 40 V.

The details of Sb_2_Te_3_ films synthesized by assisted voltages of 0 V, 20 V, 30 V, and 40 V are observed in TEM and HRTEM images, as depicted in Fig. 2. Figure 2(b,c,e,f,h,i,k,l) which originate correspondingly from the magnified image of the selected area in Figs. 2a,d,g,j. An amorphous structure of Sb_2_Te_3_ film is shown in Fig. 2a–c. It clearly shows that the disordered structure film is composed of nanoparticles which consist of lots of fine atoms group. A indistinct interface between both amorphous nanoparticles is seen and located at the dotted curve. There is no in-plane electric field applied through the substrate, that is, the assisted voltage is 0 V. The incoming particles (atoms and ions) cannot get enough momentum transfer in lateral movement on the surface for crystallization growth. The depositing particles distribute randomly on the surface of substrate, leading to the disordered formation of atoms group. For the assisted voltage of 20 V, the incoming particles of sputtering suffer electric field force in the electric field. The electric field force causes the particles to get some momentum for partial crystallization growth, at the same time, there exists still the amorphism of some atoms group (see Fig. 2e). A obvious interface between both nanoparticles is located at the dotted curve, as shown in Figs. 2f and S1a, the angle of ∼20° for intraplanar grain boundaries between (0 1 5) and (0 1 5) planes is found in the film. When the assisted voltage becomes large to 30 V, the growing particles can get more efficient momentum to migrate to the preferred sites for crystallization growth. Figure 3h shows that the film grows along the preferred [0 1 5] direction, and a perfect interface between both (0 1 5)-preferred nanoparticles is clearly seen in Figs. 2i and S1b, and there exists the angle of ∼30° for intraplanar grain boundaries between (0 1 5)-oriented planes in the film. It shows that the crystal growth needs moderate kinetics. With further increasing the assisted voltage to 40 V, the (0 1 5)-preferred plane has lost a little dominance, the structure collapse a bit occurs in the film due to too strong electric field, a number of dislocations and defects exist in the film, and the angle of ∼40° for intraplanar grain boundaries between (0 1 5) planes is shown in Figs. 2k,l and S1c. The interface between both nanoparticles implies that the growth of the particles has been into each other in high electric field. It indicates that the formation of the ordered structure is in need of the proper intensity of electric field in the Sb_2_Te_3_ films, and the angle of intraplanar grain boundaries between (0 1 5) planes seems to increases with the increasing electric field.

The morphologies of Sb_2_Te_3_ films are further studied by SEM, as shown in Fig. 3. From Fig. 3a,b, SEM images reveal that the disordered structure Sb_2_Te_3_ film has been prepared under no assisted voltage. It shows that the film is composed of numerous nanoparticles which distribute randomly in the film. Seen from the top and cross-sectional view (Fig. 3c,d), the size of particles becomes large along the electric field direction compared with the above disordered structure film, which gives direct information of the crystal structure change induced by the assisted voltage of 20 V. It further proves that the particles can get efficient momentum transfer in lateral movement on the surface for particles growth. With increasing the assisted voltage to 30 V, the size of particles further becomes larger along the electric field direction in the film which is densely grown along their oriented direction, as shown in Fig. 3e,f. The growth kinetics is influenced by the in-plane electric field which plays a key role in control over the ordered crystal structure growth. When the assisted voltage enhances to 40 V, the film seems to experience high temperature. The microstructure of all particles in the film is somewhat similar to the particles that once melted due to relatively high electric field (see Fig. 3g,h). It is fully consistent with the XRD and TEM results above.

**Figure 4:**
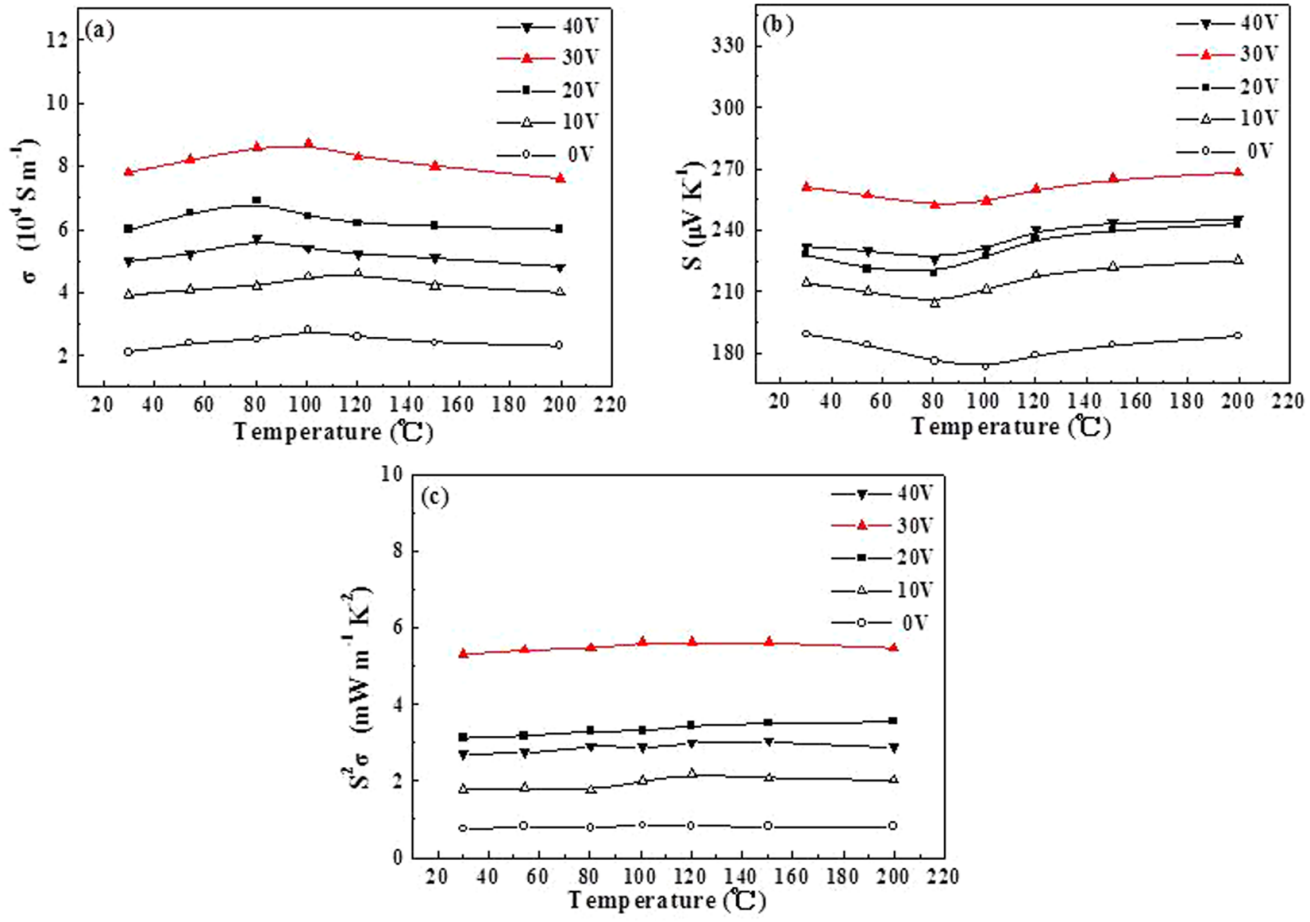
Thermoelectric performance of Sb_2_Te_3_ films deposited under different electric fields (0, 10, 20, 30 and 40 V) as a function of temperature: (**a**) *σ*, (**b**) S and (**c**) *S*^2^*σ*.

It finds that the modifying microstructure can tune the electronic transport property of materials. The *σ* values of Sb_2_Te_3_ films are examined in the in-plane direction. The *σ* values of all films are gradually increasing then decreasing in the temperature range of 30–200 °C, showing a weak temperature dependence (see Fig. 4a). The grain boundary potential barrier of Sb_2_Te_3_ films is responsible for the phenomenon. It supports an efficient application at a relatively high temperature. As shown in Fig. 4a, Sb_2_Te_3_ films fabricated by electric field voltages of 0 V, 10 V, 20 V, 30 V, and 40 V have maximum *σ* values of 2.8 × 10^4^ S m^−1^, 4.6 × 10^4^ S m^−1^, 6.9 × 10^4^ S m^−1^, 8.7 × 10^4^ S m^−1^ and 5.7 × 10^4^ S m^−1^, respectively. It is obvious that the *σ* values of films are greatly related to its microstructure. The maximum *σ* value of the ordered structure film is over 3.1 times compared with that of the disordered structure film. And it is higher than that of the films fabricated by voltages of 10 V, 20 V, 40 V and reported materials^24–26^. We consider that the 30° angle of intraplanar grain boundaries between (0 1 5)-oriented planes and the perfect interfaces between nanoparticles provide the effective channel for carriers transport, which promotes enormously increased electrical conductivity and carrier mobility in the ordered structure film.

The *S* values of Sb_2_Te_3_ films are presented along the in-plane direction in Fig. 4b, which indicate a *p*-type semiconductor. The highest *S* value reaches to 268 μV K^−1^ for the ordered structure film, which is much higher in comparison to those of other Sb_2_Te_3_ films in the present work and the reported results of (Bi,Sb)_2_(Te,Se)_3_ materials^27–31^. Why can the Seebeck coefficient be increased in the ordered structure film? It is the fact that the greatly change in the structure compared to the reported structure. There exist the angular intraplanar grain boundaries between (0 1 5)-oriented planes and the perfect interfaces, which can preserve the high quality ordered channel. Therefore, the ordered channel structure can effectively guarantee the carrier mobility, leading to a relatively high *μ*. It indicates that the hole concentration (*n*) of the film decreases to the relatively optimized level and the relaxation time (*τ*) possibly becomes large due to the ordered channel structure, resulting in a large *S*. Figure 4c gives the power factor *S*^2^*σ* results of all Sb_2_Te_3_ films. It exhibits that the maximum *S*^2^*σ* is 5.62 mW/m·K^2^ in the ordered structure Sb_2_Te_3_ film at 150 °C, which is greatly improved in compared with the Sb_2_Te_3_ films prepared by voltages of 0 V, 10 V, 20 V, 40 V and previous results^27–32^. There is no doubt that the angular intraplanar grain boundaries between (0 1 5)-oriented planes significantly aid in achieving enhanced performance.

**Figure 5:**
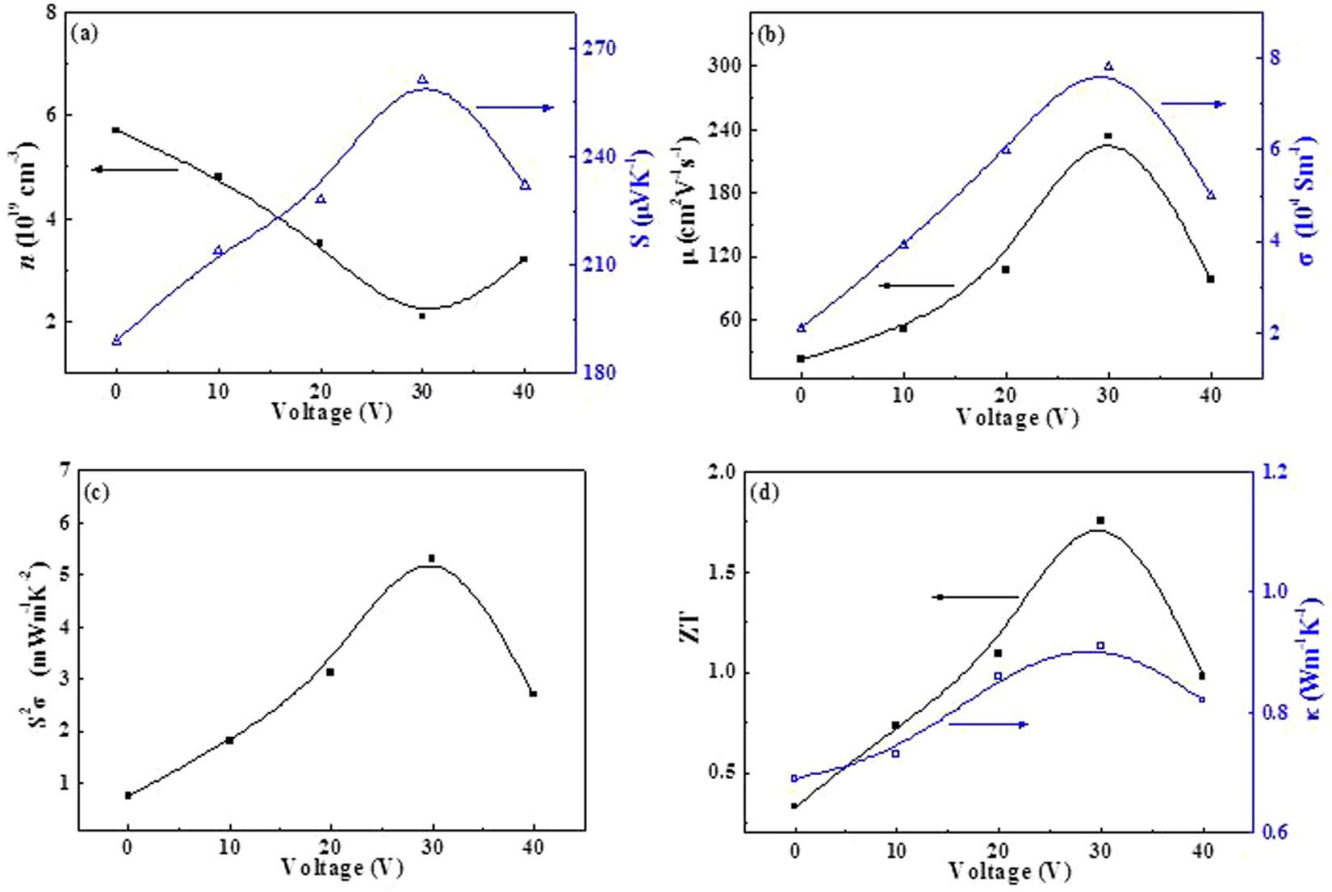
Thermoelectric properties of Sb_2_Te_3_ films deposited under different electric fields (0, 10, 20, 30 and 40 V) at room temperature: **(a**) *n* and *S*, (**b**) *μ* and *σ*, (**c**) *S*^2^*σ* as well as (**d**) *κ* and Z*T*.

Figure 5 plots the room-temperature thermoelectric performance of the deposited Sb_2_Te_3_ films. Figure 5a shows that all holes concentration of Sb_2_Te_3_ films are the value of ∼10^19^ cm^−3^. ref. ^33^. shows that the optimal carrier concentration is approximately 10^19^ cm^−3^ in (Bi,Sb)_2_(Te,Se)_3_ materials. The *μ* has a dramatic fluctuation (Fig. 5b). Particularly, Sb_2_Te_3_ films at 30 V has the highest *μ* of ~230 cm^2^ V^−1^ s^−1^, which is over double than those of others, indicating the weakened phonon-hole scattering^34^. Dominated by high *μ* of the 30 V film, the corresponding *σ* is also highest among all the as-deposited films (Fig. 5b). Other than high *σ* of the 30 V film, as shown in Fig. 5a, its *S* is also higher than others due to enhanced *μ*. As shown in Fig. 5c, contributed from the simultaneous enhancement in *σ* and *S*, the 30 V electric field deposited Sb_2_Te_3_ film has a high *S*^2^*σ* of ~5.31 mW/m·K^2^ at 300 K. The *κ* (Fig. 5d) exhibits greatly low value. Subsequently, mainly deriving from increased electrical performance, the Z*T* value (*T* = 300 K) of the 30 V deposited Sb_2_Te_3_ film can be calculated as high as 1.75 (Fig. 5d). It is also great superior to the reported results of (Bi,Sb)_2_(Te,Se)_3_ materials^35–37^, which clearly demonstrates the accurate control via external electric field during magnetron sputtering deposition process could lead to significantly high Z*T* values in the Sb_2_Te_3_ films.

**Figure 6:**
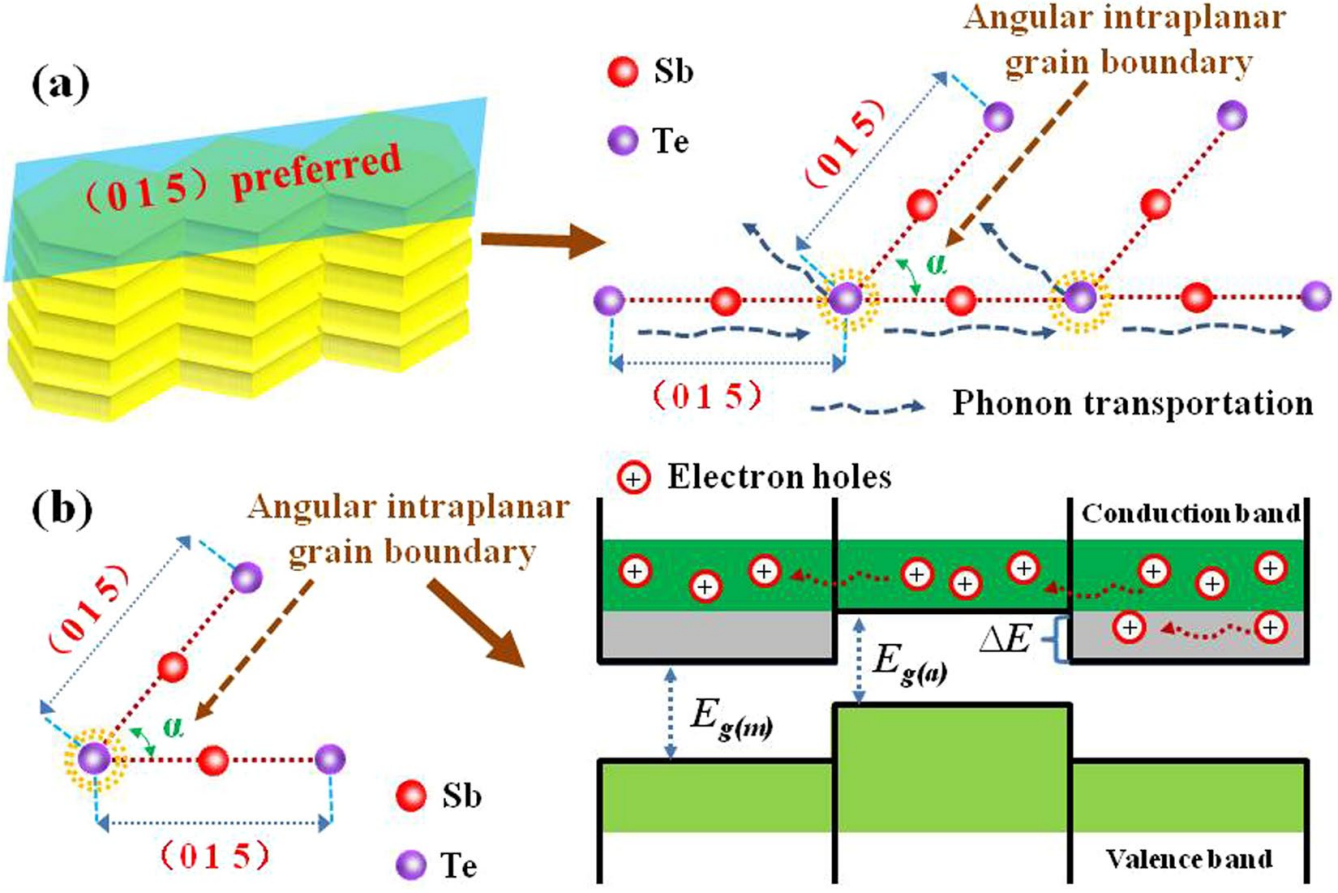
(**a**) Schematic diagram of angular intraplanar grain boundaries (between (0 1 5) and (0 1 5)) and corresponding strain fields could effectively scatter phonons. (**b**) Schematic diagram of the energy filtering effective at angular intraplanar grain boundaries.

Figure 6a schematically illustrates the external electric field-induced the surfaces of the deposited Sb_2_Te_3_ films. With increasing the electric field, the intraplanar grain boundaries between (0 1 5) and (0 1 5) planes are formed. Meanwhile, the angle between (0 1 5) and (0 1 5)-preferred surface increases as well. With such a structural change, Fig. 6a shows that additional phonon scattering centers might happen at the intraplanar grain boundaries and corresponding stronger strain fields, leading to decreased *κ*. At the intraplanar grain boundaries within the (0 1 5)-preferred surface, the angle of intraplanar grain boundaries would increase with increasing electric-field. In Fig. 6b, the additionally energy filtering effect might be introduced by such angular intraplanar grain boundaries. The energy filtering takes place at the angular intraplanar grain boundaries, which is similar to the results of grain boundaries^38^, superlattice interfaces^39^, and nanowire interfaces^40^, leading to increasing hole energy^7,10,41^. The corresponding results show that phonon-hole scattering is weakened and *μ* is correspondingly increased. Besides, *S* is enhanced as a result of the flattened band at a specific tilting angle^42,43^.

## Conclusions

A simple electric-field-assisted magnetron-sputtering deposition method is employed to control the grain boundaries of Sb_2_Te_3_ film in this work. The electric field can induce the change in the angle of intraplanar grain boundaries between (0 1 5) and (0 1 5) planes. The low *κ* value can be achieved along the in-plane direction. Meanwhile, *μ* is enhanced due to energy filtering effect. Subsequently, under 30 V external electric-field-assisted deposition, a greatly high Z*T* value of 1.75 at room temperature is observed in the ordered structure Sb_2_Te_3_ film. This work indicates that the grain-boundary control of Sb_2_Te_3_ films can be realized by external electric-field-assisted magnetron-puttering deposition method, forming angular grain boundaries and enhancing Z*T* values.

## Methods

In this work, all Sb_2_Te_3_ films are grown on conductive glass substrates by external electric-field-assisted magnetron-sputtering deposition method. To tune deposition of Sb_2_Te_3_ films, external electric fields with the voltage of 0, 10, 20, 30 and 40 V are applied through the substrate along the in-plane direction. The film deposition direction is perpendicular to the electric field direction. Commercial 60-mm-diameter hot-pressed Sb_2_Te_3_ (99.99% purity) target (purchased from General Research Institute for Nonferrous Metals, China) is used for sputtering. The Sb_2_Te_3_ target is connected to a direct-current power supply with power of 20 W. The deposition temperature is 150 °C, the base pressure is lower than 2 × 10^−4^ Pa, and the working pressure of argon is fixed at 1 Pa for all films. The thicknesses of all films are ~1.5 μm by adjusting the deposition rate and sputtering time in experiments.

Crystal structures of all films are characterized by XRD (Rigaku D/MAX 2200) with Cu Kα source (λ = 0.154056 nm). The morphologies of as-deposited films are investigated using a field emission SEM (Sirion 200). Further structural analyses are performed by TEM (FEI Company, Tecnai G2 F20S-Twin FEG TEM at 200 kV). A surface profilometry (Ambios XP-2, USA) is used to measure the controllable film thickness. The *S* and *σ* of as-deposited films along in-plane directions are measured by a ZEM-3 system (Ulvac Riko) with a homemade holder. Three repeated measurements on the samples did not show any degradation, the variations were within a few percent for the samples made by the same procedure (see Figs. 4 and S2). A Laser PIT (Ulvac Riko) is used to collect the in-plane *κ* data at room temperature. The 1.5-μm-thick film of Sb_2_Te_3_ was deposited on a specially designed specimen-holder frame substrate. The substrate and frame materials are borosilicate glass having a thickness of 30 μm and 200 μm, respectively, supplied by NIMS (Xu Group, Japan). Each sample was repeatedly measured at least three times by Xu Group to achieve a thermal conductivity value. The recent references^14,44^ shows the principle of the measurement method in detail. The errors for Seebeck coefficient, thermal conductivity, and electrical conductivity are 5%, 4%, and 4%, respectively. The Hall measurement system (ECOPIA HMS-3000) is used to determine the *n* and *μ* at room temperature.

## Supplementary information

Supplementary information
