# Supplementary material for: Approaching high-performance of ordered structure Sb2Te3 film via unique angular intraplanar grain boundaries
Source: Sci Rep. 2020 Apr 6;10:5978. doi: 10.1038/s41598-020-63062-z (PMC7136274; doi:10.1038/s41598-020-63062-z)
Supplement: Supplementary file 1 — Supplementary information [file 41598_2020_63062_MOESM1_ESM.doc]

**Supporting Information**

**Approaching high-performance of ordered structure** **Sb_2_Te_3_ film via unique angular intraplanar grain boundaries**

Ming Tan, Liyu Hao, Hui Li, Cong Li, Xiaobiao Liu, Dali Yan, Tie Yang, Yuan Deng

**Table S1** The sizes of grains in the Sb_2_Te_3_ films.

| Voltage of electric field (V) |  | Grain size (nm) |
| --- | --- | --- |
| 0  10  20 |  | 27.2  39.5  59.1 |
| 30 |  | 80.2 |
| 40 |  | 91.0 |


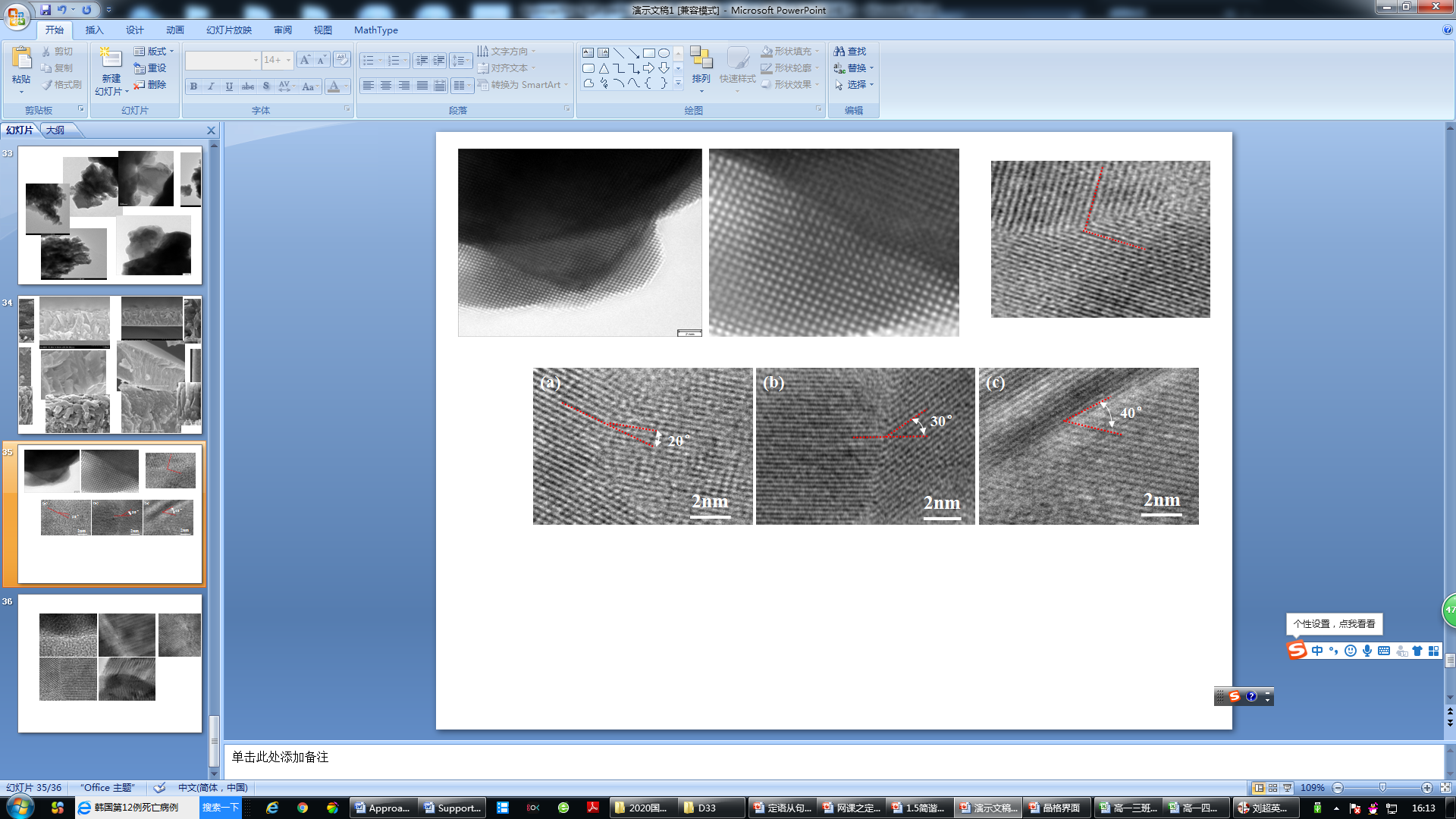


**Fig. S1** TEM images of grain boundaries of Sb_2_Te_3_ films prepared by assisted voltages of (a) 20 V, (b) 30 V, and (c) 40 V, respectively.


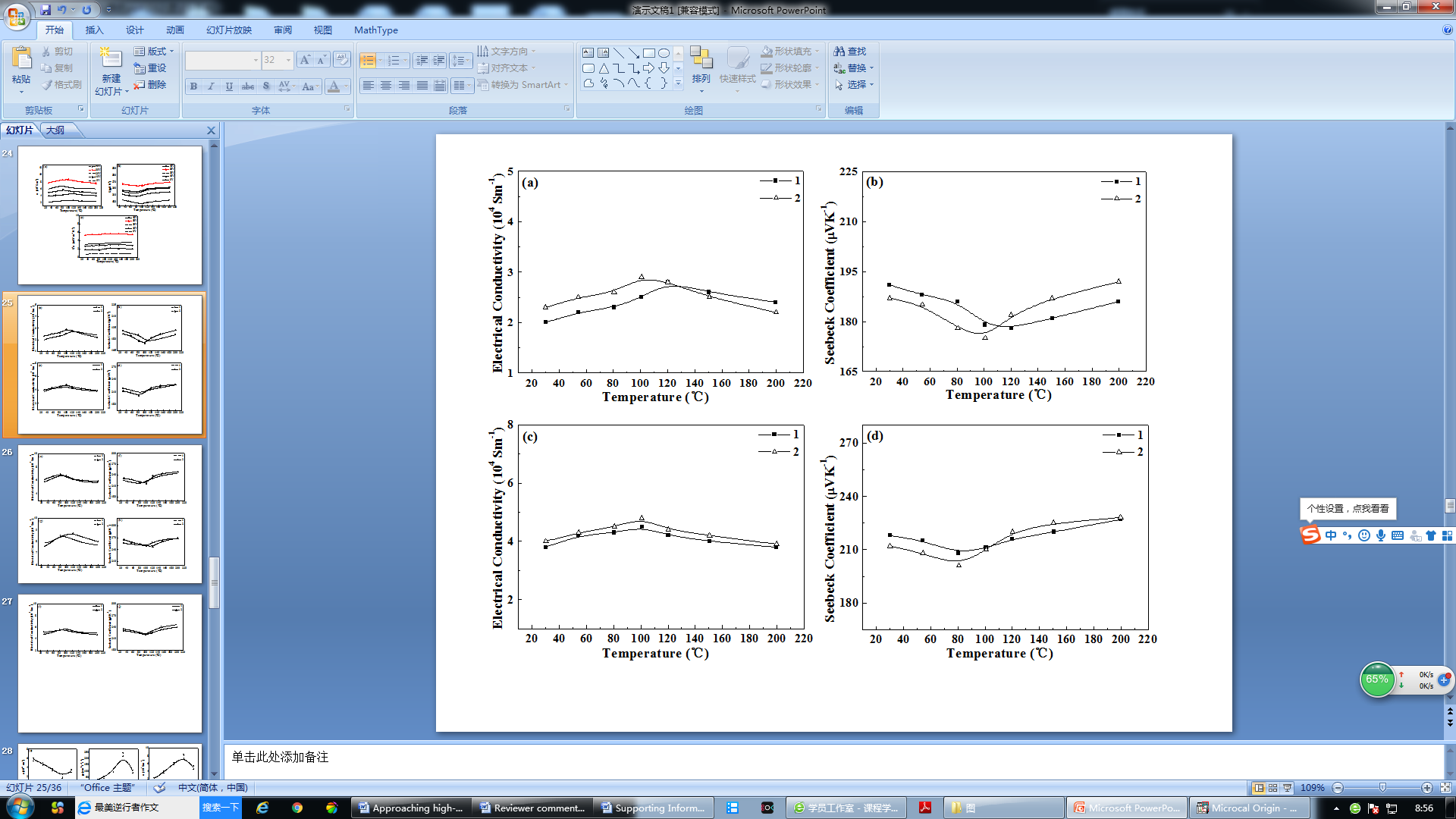


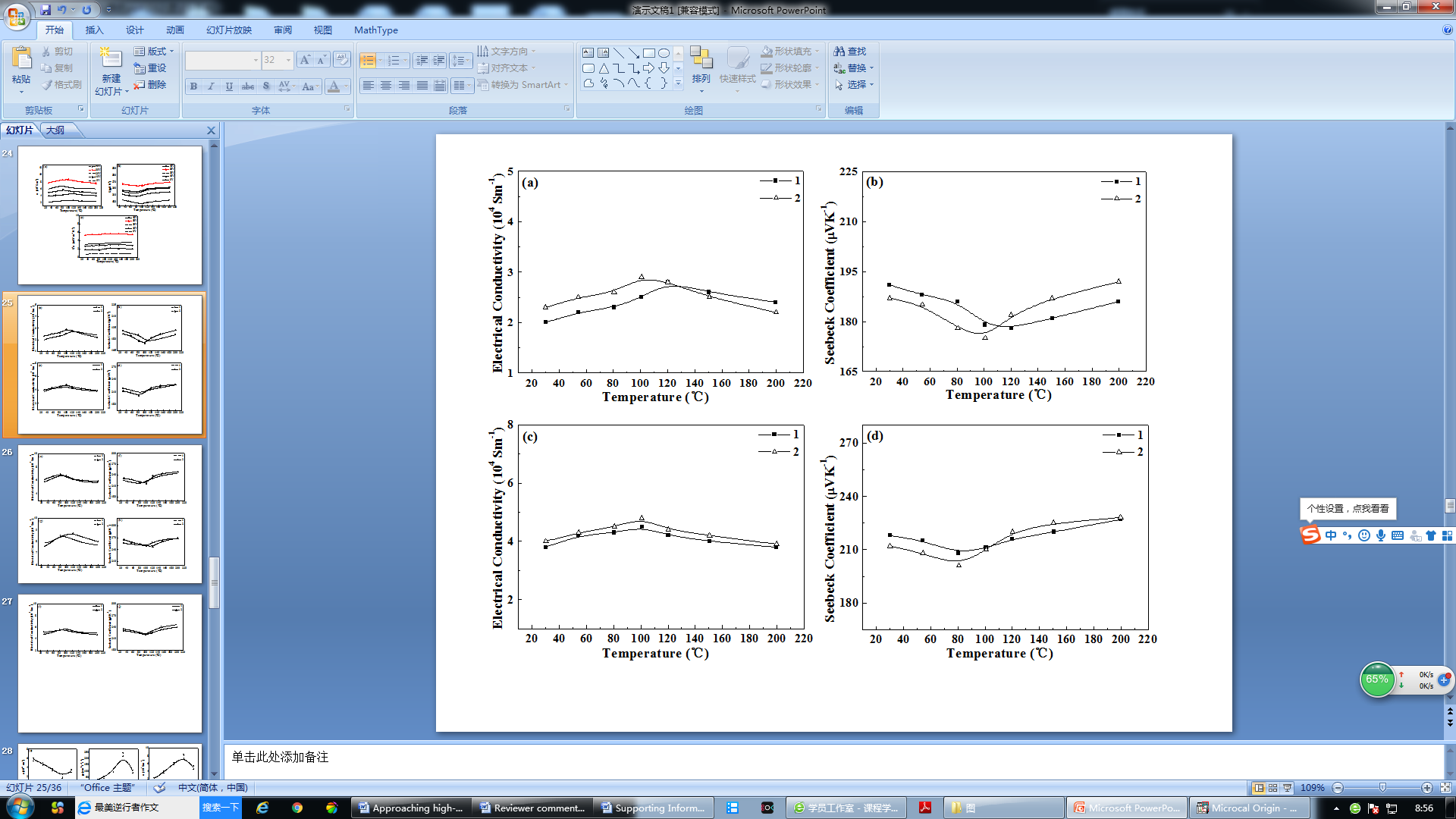


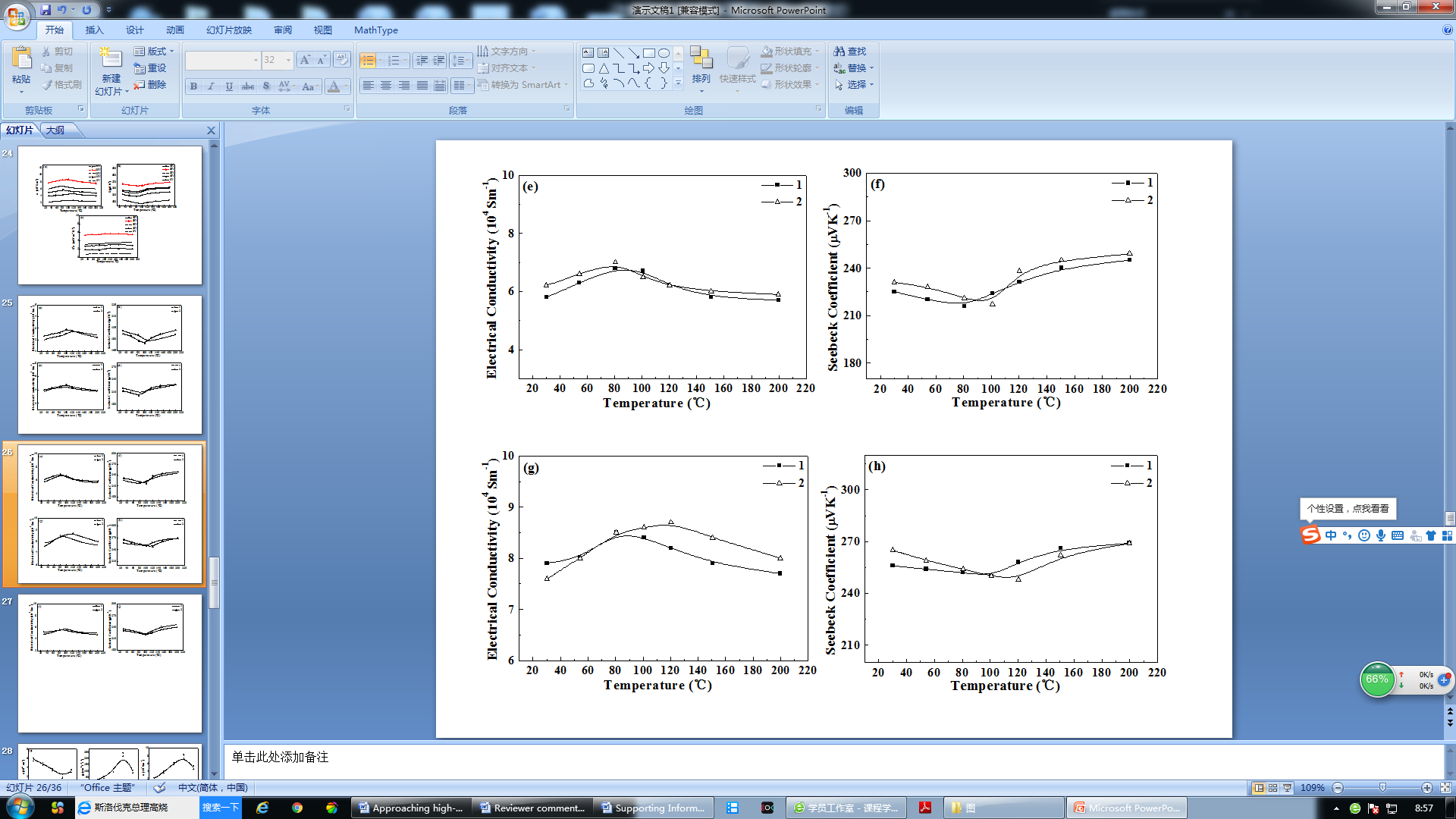


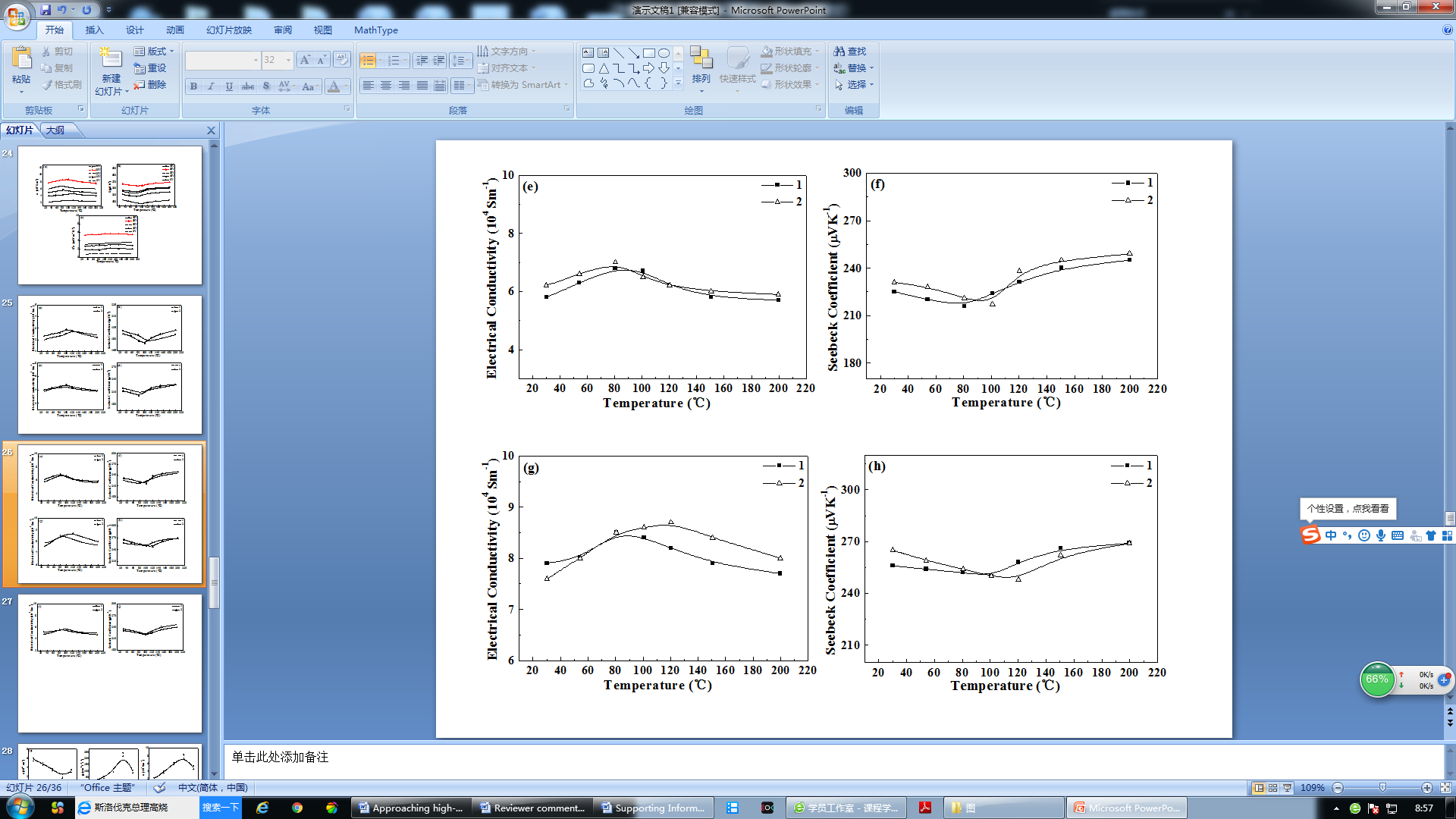


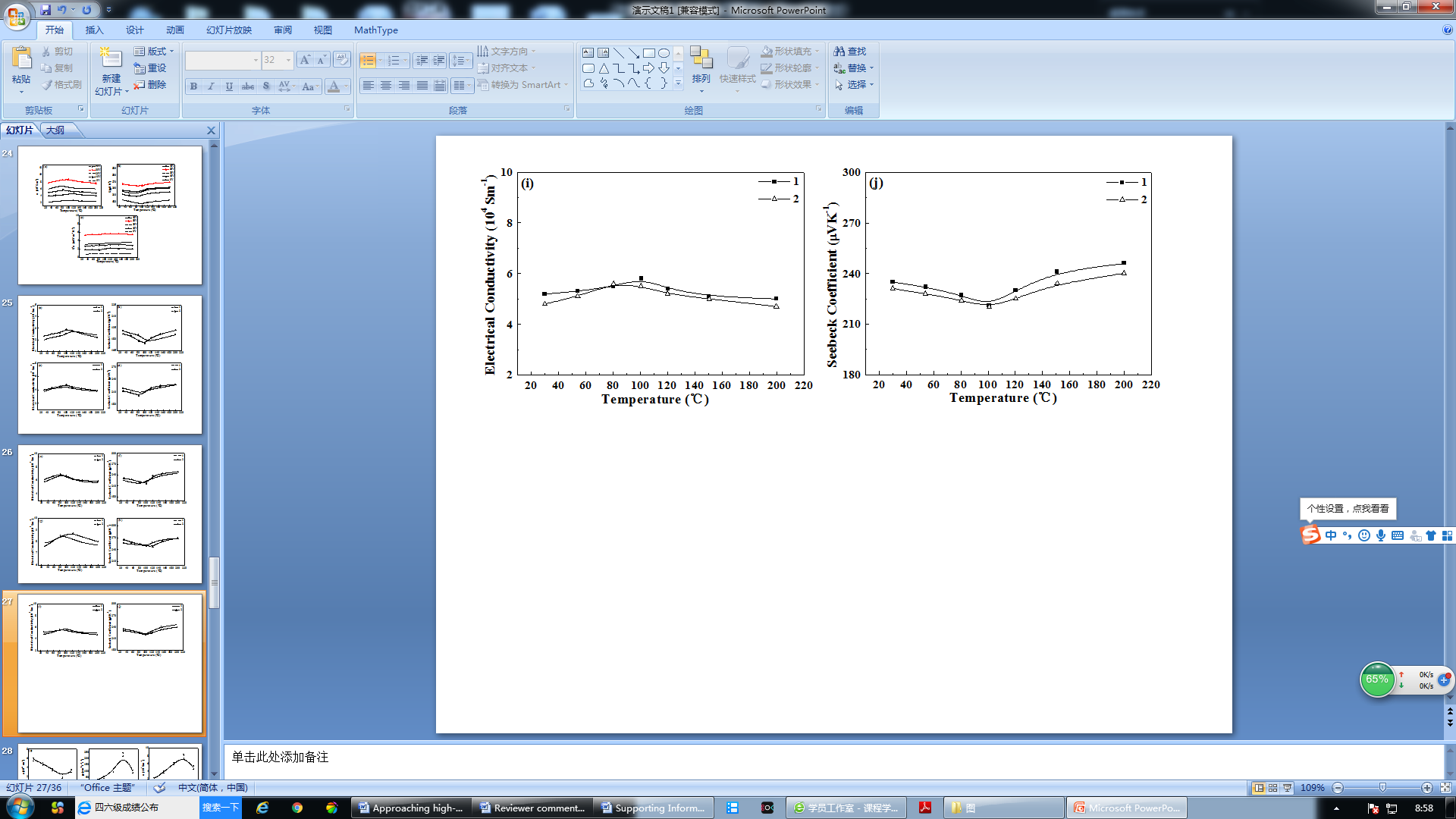


**Fig. S2** Thermoelectric performance of Sb_2_Te_3_ films deposited under 0, 10, 20, 30 and 40 V electric fields, respectively, as a function of temperature: (a,c,e,g,i) *σ* and (b,d,f,h,j) *S*. (They are another two repeated measurements’ results of each sample.)
